# A Novel Flexible Virtual Fixtures for Teleoperation

**DOI:** 10.1155/2014/897242

**Published:** 2014-02-11

**Authors:** Guanglong Du, Ping Zhang

**Affiliations:** South China University of Technology, Guangzhou 510000, China

## Abstract

This paper proposed a novel spatial-motion-constraints virtual fixtures (VFs) method for the human-machine interface collaborative technique. In our method, two 3D flexible VFs have been presented: warning pipe and safe pipe. And a potential-collision-detection method based on two flexible VFs has been proposed. The safe pipe constructs the safe workspace dynamically for the robot, which makes it possible to detect the potential collision between the robot and the obstacles. By calculating the speed and the acceleration of the robot end-effecter (EE), the warning pipe can adjust its radius to detect the deviation from the EE to the reference path. These spatial constraints serve as constraint conditions for constrained robot control. The approach enables multiobstacle manipulation task of telerobot in precise interactive teleoperation environment. We illustrate our approach on a teleoperative manipulation task and analyze the performance results. The performance-comparison experimental results demonstrate that the control mode employing our method can assist the operator more precisely in teleoperative tasks. Due to the properties such as collision avoidance and safety, operators can complete the tasks more efficiently along with reduction in operating tension.

## 1. Introduction

The concept of virtual fixtures is presented by Kikuuwe [1] from Stanford University in 1993 to solve the delay problem and to improve the operability of the teleoperation system. Rosenberg constructed 8 types of virtual fixtures in a peg-and-hole task and found out that they can decrease operation time at some extent and increase efficiency by 20 to 70%. Because virtual fixtures have a broad application background in teleoperation, human-machine cooperative system and medicine, fine production, and other fields, there are many thorough researches about it.

Most of these researches are focused on two types of virtual fixtures: guidance virtual fixtures and forbidden region virtual fixtures. Guidance virtual fixtures are needed when a robotic manipulator is needed to move along with a planned path precisely. Forbidden region virtual fixtures are used to prevent a robotic manipulator entering some specific region in order to avoid some damage. Bettini et al. [2] studied the performance of real-time video feedback virtual fixtures. Based on this research, Kang, Park, and Ewing studied the performance of video and tactile feedback virtual fixtures. Nowadays, with the help of some techniques such as virtual fixtures (VFs), high precise manipulation task is finished by robotic assistants. The surgeon is capable of more precise surgery in robotic-assisted procedures. VFs are algorithms which limit a robotic manipulator into restricted regions [3–7] and/or direct a robotic manipulator to move along with the planned path [8–10]. This following literature has discussed VFs. References [8, 11–13] are for telerobots and [9, 14–17] are for cooperative robots.

FRVFs (forbidden region virtual fixtures) are used to restrict the surgical tool into certain region in workspace. Beasley and Howe [18] set an active constraint to guide the robot to cut femur and tibia within a permitted region in prosthetic knee surgery. Park et al. [19] developed VFs based on sensor that limit the robot's motion or direct the surgeon to move the surgical instruments in a planned path using haptic feedback. During a teleoperated coronary bypass, there is a virtual wall guiding a surgeon's instrument based on the location of the internal mammary artery obtained from a preoperative computer tomography scan.

Bettini et al. [9] concentrated on researches for guidance VFs. They used vision information to generate VFs, examined the hard and soft VFs, and worked on the application to vitreoretinal surgery. Marayong et al. [20] demonstrated motion constraints by varying compliance that was described for the general spatial case. In these researches, admittance control laws are used to implement VFs. A passive arm with dynamic constraints (PADyc) has been developed by Li et al. [15, 17, 21] for pericardial puncture. They implement VFs to restrict surgical tools to move along planned paths or away from forbidden regions by using electrical motors to choose a clutching system for freewheels. The main advantage of this method is that the robot cannot provide motive force without the help of the surgeon. This has been considered as one safety advantage because it can prevent a robot from losing control. But there are some limitations including mechanical complexity and loss of the robot's ability to actively assist in surgical procedures. A robotic system for fully automated paranasal sinus surgery is developed by Wurm et al. [22]. This system uses preoperative CT to direct the robot's autonomous motion and it allows being remote controlled by a joy stick. A mechatronic system for FESS (functional endoscopic sinus surgery) has been presented by Lueth's group [23–25]. According to a 3D model from CT data, they planned a safe working space preoperatively. In the middle of the operation, the shaver is automatically turned on/off according to the position of the shaver tip. In the safe area, the shaver reacts to signals from the surgeon. When the tip of the shaver moves outside the safe area, an electrical pulse will stop the shaver by interrupting its automatic drive control. This navigation-based system is only concerned with the position of shaver tip.

**Figure 1:**
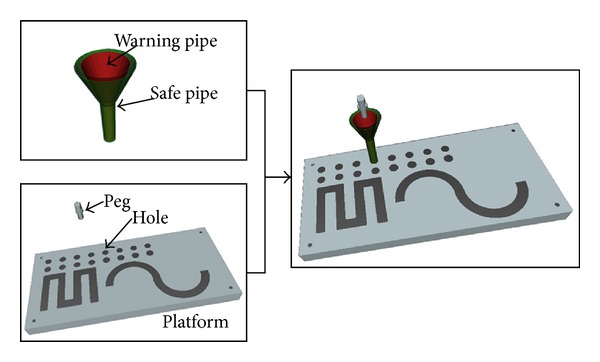
Geometric relation for spatial motion constraints.

In this paper, we present an online obstacle-avoidance method for serial robot in geometrically complex environments. We extend Li's work [15] to generate VFs for obstacle avoidance and a new potential collision-constraint-detection method has been proposed. In this method, two pipes (the warning pipe and the safe pipe) are automatically generated from 3D reference path in real time. The safe pipe serves as spatial constraints for constrained robot control. The warning pipe adjusts the radius to detect the deviation of the robot EE from the reference path by calculating the speed and the acceleration of the robot EE. In our experiment (Figure 1), two assisted modes were implemented: one pipe assisted mode [15] (OPAM) and two pipes assisted mode (TPAM). The former mode used the fixed VFs [15] to guide the robot EE but in our method (the latter mode), two types of VFs were used to guide and constrain the robot EE. When the robot EE collides with the pipes, our system will give warning information based on vision to the operator: changing the color of the pipes. Due to the warning pipe, our new spatial-motion-constraints method enables multiobstacle manipulation task of telerobot in precise interactive teleoperation environment.

The remainder of the paper is organized as follows.  Section 2 provides a brief summary of path and the pipes building algorithm. In Section 3, we introduce the dynamic adjustment algorithm of the safe pipe and the warning pipe. In Section 4, we report the experiment results of two control modes. We conclude the paper and discuss possible future extensions in Section 5.

## 2. Path and Pipes

The path is a possible route obtained by the robot path planning. Path planning is a kind of algorithm which finds out a collision-free path in all generalized coordinates of a robot with some valuation criteria by the given initial positions and orientations of the robot. A pipe gives the robot a safe space in which the robot can move freely without collision. The pipes are built based on the paths. They can divide the safe spaces and can also guide and give active early warnings.

The pipes take the paths as medial axis, and the path is formed by a series of path points. Thus, the pipes are formed by discrete pipe units.

The pipes are built by many pipe units. The pipe units are formed by two cross sections and a cylinder. The shape of cross section is decided by the predefined shape of the pipes. Cross section is formed by polygon and circle section is formed by polygon which has more sides.

### 2.1. Choosing a Starting Point of Cross Section

As we know, a polygon can be finalized by the given central point, the central axis, and the starting point. How to choose the starting point depends on the origin of the coordinate system. There is a central point *P*, a central axis *L*, and an origin point *O*. Firstly, connect the original point and the central point to get the reference vector *OP*. The vertical vector *Q* can be calculated:

(1) $$\begin{array}{c} Q=OP\times L. \end{array}$$

Unitize *Q* and then get *t*_*Q*_. *S* is the point which is *r* length from *P* along the direction of

(2) $$\begin{array}{c} S=P+t_{Q}\cdot r. \end{array}$$

When there is an overlap between the central point and the origin point, we can take the unit vector of *X* axis to be the reference vector.

### 2.2. Constructing a Polygon

The key to create a polygon is to determine the vertexes of the polygon. Assume that there is a polygon with *n* sides. Then the radius angle of two adjacent vertices is

(3) $$\begin{array}{c} \theta =2\cdot \frac{\pi }{n}. \end{array}$$

After the starting point and the central axis were known, the second point can be obtained through rotating the central axis by *θ* degrees.

Below is the transformation matrix of the next point which revolves around the previous point. Assuming that the coordinate of the previous point is **A**(*A*_1_, *A*_2_, *A*_3_), the central axis is **B**(*B*_1_, *B*_2_, *B*_3_). To revolve around **B** axis, **A** should move to the origin. Then revolves around the *θ* degrees of the central axis by *θ* degrees and move it inversely. Thus, this transformation matrix **T** is

(4) $$\begin{array}{c} T=\left[\begin{array}{cccc} m_{11} & m_{12} & m_{13} & A_{1}-m_{11}\cdot A_{1}-m_{12}\cdot A_{2}-m_{13}\cdot A_{3}\\ m_{21} & m_{22} & m_{23} & A_{2}-m_{21}\cdot A_{1}-m_{22}\cdot A_{2}-m_{23}\cdot A_{3}\\ m_{31} & m_{32} & m_{33} & A_{3}-m_{31}\cdot A_{1}-m_{32}\cdot A_{2}-m_{33}\cdot A_{3}\\ 0 & 0 & 0 & 1 \end{array}\right]. \end{array}$$

In this equation, we have

(5) $$\begin{array}{c} m_{11}=t\cdot B_{1}\cdot B_{1}+c;\\ m_{12}=t\cdot B_{1}\cdot B_{2}+s\cdot B_{3};\\ m_{13}=t\cdot B_{1}\cdot B_{3}-s\cdot B_{2};\\ m_{21}=t\cdot B_{1}\cdot B_{2}-s\cdot B_{3};\\ m_{22}=t\cdot B_{2}\cdot B_{2}+c;\\ m_{23}=t\cdot P_{2}\cdot P_{3}+s\cdot P;\\ m_{31}=t\cdot B_{1}\cdot B_{3}+s\cdot B_{2};\\ m_{32}=t\cdot B_{2}\cdot B_{3}-s\cdot B_{1};\\ m_{33}=t\cdot B_{3}\cdot B_{3}+c;\\ c=\cos\theta ;\\ s=\sin\theta ;\\ t=1-\cos\theta . \end{array}$$

The next **A**′ can be obtained by

(6) $$\begin{array}{c} A^{\prime }=T\cdot A. \end{array}$$

After determining the *n* − 1 points, the whole vertexes can be determined. Correspondingly, cross section is determined. And then next step should be the construction of cylinders.

### 2.3. Constructions of Cylinders

To enable pipes to detect collision in virtual environment, pipes are formed by triangles. In this algorithm, cylinders are formed by connecting two vertexes of two adjacent cross sections.

The rule of constructing triangles is to pick up and connect the three adjacent points in each row like {1,2, 3}, {2,3, 4},…, {*n* − 1, *n*, *n* + 1} which means the (*n* + 1)th point is completely overlapped with the first point and then that makes sure that cylinders are joined together.

### 2.4. Splicing of Pipe Units

If there are two pipe orifices which have the same shape, the algorithm of splicing pipe units is the same as above (algorithm of constructing cylinders). When the shapes of two pipe orifices are different, there need to be splicing pipe units to join those pipes. The algorithm of creating the splicing pipe is introduced below.

Assume that the shape of the cross section of the pipe 1 is a polygon which has *n*_1_ sides. And assume that for pipe 2, the cross section is *n*_2_. So there also needs to be a linking pipe to connect pipe 1 and pipe 2. This paper chooses *n*_3_ sides polygon; here *n*_3_ is the common multiple of *n*_1_ and *n*_2_ as the cross section of the linking pipe

(7) $$\begin{array}{c} n_{3}=gcd\left(n_{1},n_{2}\right). \end{array}$$

Here *gcd* means the common multiple of the two numbers.

When connecting two pipes, the triangles' numbering rule needs to be adjusted because the shapes of two polygons are different. As we know, *n*_3_ can be divisible by *n*_1_ or *n*_2_. When numbering the triangles, many-to-one mapping strategy is employed.

## 3. Dynamic Pipe Adjustment Algorithm

Pipes which are generated by this generation algorithm can be changed dynamically according to the given path and radius. Here are two dynamic pipe adjustment algorithms. The first one is dynamic pipe scaling algorithm. It generates the extended safe pipe which plays a guide role and divides safe region. The robot EE can reach its destination safely as long as it does not exceed the range of pipe region and moves along the direction of the pipe.

The second one is pipe early warning method. This method would generate two pipes. The first pipe is the safe pipe which cannot be extended dynamically but can guide the operators and provide the safe region. The second pipe is the early warning pipe. It gives a real-time warning once there was any muscles jitter of the operators during the robot controlling.

### 3.1. Dynamic Extension Algorithm

**Figure 2:**
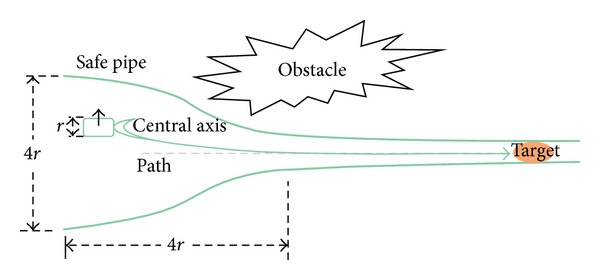
Result of dynamic safe pipe adjustment.

Safe extended pipes need to be adjusted dynamically according to how far the robot EE strays from the path. One capability of the safe extended pipes is to provide a safe region to protect robot EE from colliding with other objects as long as it moves within the safe region (Figure 2). That requires the safe extended pipes to adjust dynamically and to give the operator an early warning. The safe pipe region should be big enough for the robot EE to move around. Safe extended pipe keeps extending until it reaches around the obstacles. If the robot EE crosses the safe extended pipe, The system will issue a warning that the robot EE is in a dangerous region. Safe extended pipes also functioned as guidance. In this method, safe extended pipe is designed as a pipe from thick to spindly which can lead the robot EE to move towards the target.

Given a safe path, the robot EE is at the starting point of the safe path. Assume that the diameter of the robot EE's cross section is *k*, the length of the path is *G*, and also *G* > 4*k*. The initial point is *P*_0_ and *P*_4*k*_ is the point which is 4*k* away from *P*_0_. At *P*_0_ the cross section diameter of the safe extended pipe is 4*k* and at *P*_4*k*_ it changes to 2*k*. Thus, for any point *P*_*n*_, the cross section cross-sectional diameter is

(8) $$\begin{array}{c} d=\{\begin{array}{cc} 4k-\frac{\left|P_{n}P_{0}\right|}{4k}\cdot 2k, & \left|P_{n}P_{0}\right|<4k,\\ 2k, & \left|P_{n}P_{0}\right|>4k. \end{array} \end{array}$$

When the robot EE strays from the safe path, the safe extended pipe needs to be adjusted accordingly to fit the changed working space of the robot EE. To display the direction and the rate of deviation of the robot EE intuitively, dynamic self-adaptive adjustment strategy is employed. Here we take the extended direction of the safe pipe to be *Y* and the extended amount to be *D*.

Assume that robot EE strays from safe path in *y* direction and its offset is *x*. According to the self-adaptive adjustment strategy, the cross-sectional centre of the safe extended pipe at the initial point is the centre of the robot EE and the cross-sectional radius is 2*k* + *x*. Thus, the other centre points of the safe extended pipe *P*′are adjusted as follows:

(9) $$\begin{array}{c} P^{\prime }=\{\begin{array}{cc} P_{c}+\left(x-\frac{\left|\overset{⟶}{P_{c}P_{0}}\right|}{4k}\cdot \left(x\right)\right)\cdot \delta , & \left|P_{c}P_{0}\right|<4k,\\ P_{c}, & \left|P_{c}P_{0}\right|>4k. \end{array} \end{array}$$

Here *δ* is a unit normal vector and *P*_*c*_ is the original center point of the safe extended pipe.

Cross-sectional's radius of *P*′ is adjusted as follows:

(10) $$\begin{array}{c} d=\{\begin{array}{cc} \left(4k+2x\right)-\frac{\left|P^{\prime }P_{0}\right|}{4k}\cdot \left(2k+2x\right), & \left|P^{\prime }P_{0}\right|<4k,\\ 2k, & \left|P^{\prime }P_{0}\right|>4k. \end{array} \end{array}$$

### 3.2. Initiative Early Warning Algorithm of Safe Pipes

**Figure 3:**
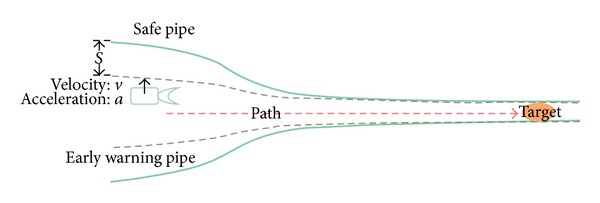
Result of dynamic early warning pipes adjustment.

In the initiative warning algorithm, there are two pipes. The outer one is a safe pipe and the inner one is an early warning pipe. Formula (8) can be a reference to the generation algorithm of safe pipe. The difference between the safe pipe and the safe extended pipe is that the safe pipe cannot be extended dynamically. Early warning pipe is used to monitor the deviation of the robot EE and it needs to be adjusted according to the deviation of the robot EE (Figure 3). If the robot EE crosses warning pipe, the system will alarm a warning report operator that the robot EE is in dangerous region. The safe extended pipe is designed as a pipe from thick to spindly which can lead the robot EE to the target.

Given a path, the robot EE is at the initial point of the path. Assuming that the cross-sectional diameter is *h*, the length of the path is *R* and *R* > 4*h*. The initial point is *C*_0_ and *C*_4*h*_ is the point which is 4*h* away from *P*_0_. The cross-sectional diameter at *C*_0_ is 4*h* and the diameter is *h* when at *P*_4*h*_. Thus, for any point *C*, its cross-sectional diameter is

(11) $$\begin{array}{c} d_{s}=\{\begin{array}{cc} 4h-\frac{\left|CC_{0}\right|}{4h}\cdot 2h, & \left|CC_{0}\right|<4h,\\ 2h, & \left|CC_{0}\right|>4h. \end{array} \end{array}$$

When the robot EE strays from the path, the early warning pipe needs to adjust itself accordingly, so that it can give a real-time warning when the robot EE crosses safe pipe.

Assuming that the robot's current moving speed is *v*, its acceleration is *a*. Then the robot's displacement at the next moment is

(12) $$\begin{array}{c} s=vt+\frac{1}{2}at^{2}. \end{array}$$

Here *t* is time interval.

The diameter of early pipe is

(13) $$\begin{array}{c} d_{w}=\{\begin{array}{cc} \left(4h-2s\right)-\frac{\left|CC_{0}\right|}{4h}\cdot \left(2h-2s\right), & \left|CC_{0}\right|<4h,\\ 2h, & \left|CC_{0}\right|>4h. \end{array} \end{array}$$

### 3.3. Collision Detection

In order to detect whether the robot EE passes through the pipe, this paper uses K-DOPs algorithm [26] to detect the collision between the robot EE and the pipes. K-DOPs can detect the collision in real time. In order to use K-DOPs, the pipe is designed as a set of triangles. In addition, K-DOPs can calculate the collision point and the collision direction. When the robot EE passes through the pipe, K-DOPs will calculate the crossing position and the direction, and then the operator can adjust the robot EE to the negative direction.

### 3.4. Analysis of Time Complexity

Assuming the sides of the pipe units are *n*′ and the number of discrete points of the pipe path is *m*, then the frequency of choosing initial points is *m*, the frequency of constructing polygon is 16*n*′*m*, the frequency of constructing cylinder is *m*(*n*′ − 1), and the frequency of connecting pipe units is 3*n*′(*m* − 1). Thus, the total frequency of constructing pipes is

(14) f(n′)=m+16n′m+m(n′−1)+3n′(m−1)=20mn′−3n′.

Its time complexity is

(15) $$\begin{array}{c} T\left(n^{\prime }\right)=O\left(mn^{\prime }\right). \end{array}$$

In the dynamic pipes adjustment algorithm, radius of each pipe unit is only updated once. And for every point in the pipe, its coordinates are needed to be recalculated once. Then the total frequency is

(16) $$\begin{array}{c} f_{1}\left(n^{\prime }\right)=16n^{\prime }m. \end{array}$$

Its time complexity is

(17) $$\begin{array}{c} T_{1}\left(n^{\prime }\right)=O\left(mn^{\prime }\right). \end{array}$$

## 4. Evaluation

Considering of all the described pipes and their effect, a series of tests are proposed to evaluate this teleoperation assistance. The test is performed on grabbing an object from a cabined box in a 3D environment. The experiments were carried out by 6 individual computer experts, who were men and women between 22 and 30 years old.

### 4.1. Experimentation Environment

**Figure 4:**
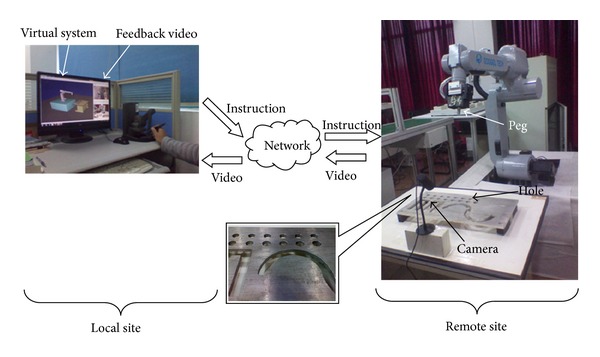
System structure.

A teleoperation platform based on virtual reality is built up (see Figure 4). In the local site, a virtual emulator system (VES) and a video feedback system (VFS) are built up to feed back the information to the operator. The video is serial images which are from the cameras fitted in the remote site. The remote cameras are used to watch the state of the real robot. In this experiment, considering the real environment of teleoperation, the system limits bandwidth to 30 kB/s and the delay time is approximately 3 seconds. The robot is a GOOGOL GRB606 with 6 DOF. Firstly, the teleoperated robot is about 80 cm from the table. The aim of the task is inserting the peg into the hole without colliding with the table. A 6-DOF force feedback device (FFD, PHANTOM DESKTOP) is used as the input interface device so that the operator can move and orient the robot EE. A camera with a resolution of 640 × 480 pixels is mounted to feed the visible scene back to the operator as the output interface device (OID). The feedback and the virtual display consist of a 16.1 in, 1280 × 1024 pixel resolution monitor. The peg was cylinder with 7.5 mm in radius and 50 mm long. The radius of the hole was 19 mm. The robot EE consisted of a square block and two claws. The size of the block was 100 mm (*W*) × 100 mm (*L*) × 80 mm (*H*) and that of the claw was 100 mm (*W*) × 100 mm (*L*) × 10 mm (*H*). The OID programs run on an Intel(R) Core(TM) PC.  Figure 7 shows the experimental environment. The workspace of FFD was 160 mm (*X*) × 130 mm (*Y*) × 130 mm (*Z*) and the position resolution of it was 0.2 mm.

### 4.2. Grabbing Object Experiment

There are two modes to carry out the task: two pipes assisted mode (TPAM) and one pipe assisted mode (OPAM) [15]. In the local site, a virtual system and a video feedback system were designed to assist the operator. The operator can make serial safe movements in the virtual system and send the instruction to the telerobot. The operator can carry out the task by continuous instructions. When there is any collision between the virtual pipes and the virtual box, the operator must make all the instructions over again.

In OPAM, a fixed VF [15] was designed to assist the operator to insert the peg into the hole. The fixed VF was a symmetrical pipe and one end of it is thick but the other one is thin. The radius of the one end closed to the robot EE was 100 mm, and the other end closed to the hole was 19 mm. The operator could control the FFD to move and orient the robot EE to control the peg along with the pipe. In this mode, the operator monitored the virtual window and the feedback video window to catch the state of the remote robot. When the robot EE was close to the hole, the operator must be very careful because the robot EE would have collided with the box easily.

In TPAM, two pipes were designed automatically to help the operator to locate the robot EE without any collision. In our method, we did not need to design the pipes. The thing we needed to do was set the path. In the experiment, we drew a safe path from the EE to the center of the hole, and then the system created two pipes automatically: the safe pipe and the warning pipe. The safe pipe could expand until the safe pipe collided with the edge of the hole and the warning pipe would adjust the radius of itself. In this mode, the operator monitored the virtual window and the feedback video window to catch the state of the remote robot. As the operator moves the robot toward the hole, the safe pipe changes continuously to make sure the robot EE will not collide with the edge of the hole.

There were two steps in each experiment: approaching and inserting. In the period of approaching, the workspace of the robot was 800 mm × 800 mm × 800 mm but in the period of inserting, it was 80 mm × 80 mm × 80 mm. Since the workspace of FFD was 160 mm (*X*) × 130 mm (*Y*) × 130 mm (*Z*) and the position resolution of it was 0.2 mm, the accuracy of the robot control was 1.10 mm (*X*) × 1.23 mm (*Y*) × 1.23 mm (*Z*) and 0.10 mm (*X*) × 0.12 mm (*Y*) × 0.12 mm (*Z*) in the period of approaching and inserting, respectively. The experiments were carried out by 6 individual operators, who were men and women between 23 and 30 years old.

### 4.3. Result

**Figure 5:**
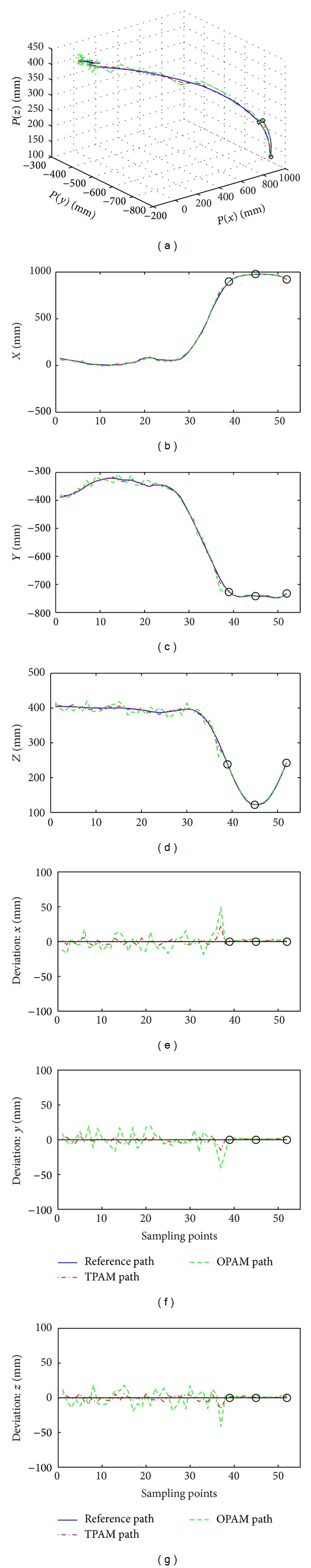
Analysis of the experiment.

The results of the peg-into-hole under TPAM and OPAM are shown in Tables 1 and 2 and Figure 5 (Test 3). The 3D paths of the robot EE in TPAM and OPAM as well as the reference path for the entire test 3 are shown in Figure 5(a). Figures 5(b)–5(d) show the displacements of the robot EE positions. There are three circles on each curve of the reference paths. From the beginning to the first circle is the period of closing to the hole, from the first circle to the second circle is the period of inserting the peg, and from the second circle to the third circle is the period of departing from the hole. The period of closing to the hole was from 1st s to 9th s, and the period of inserting the peg into the hold was from 9th s to 13th s, as well as the period of departing from the hole was from 13th s to the 17th s.

In order to evaluate the goodness of the path, the deviation from the reference path was introduced as path errors.

Supposing that the sampling point of the reference path is *W*_*k*_(*x*_*k*_, *y*_*k*_, *z*_*k*_), and *V*_*k*_(*x*_*k*_′, *y*_*k*_′, *z*_*k*_′) is the sampling point of the practice path, where *k* = 1, 2,…, *N* and *N* is the number of the sample points, the path errors can be defined as follows.

The deviation between two paths in *x*, *y*, *z* is

(18) $$\begin{array}{c} e_{x}=\frac{\left(\sum_{k=1}^{N}||x_{k}-x_{k}^{\prime }||\right)}{N},\\ e_{x}=\frac{\left(\sum_{k=1}^{N}||y_{k}-y_{k}^{\prime }||\right)}{N},\\ e_{x}=\frac{\left(\sum_{k=1}^{N}||z_{k}-z_{k}^{\prime }||\right)}{N}. \end{array}$$

And the 3D path error is defined as follows:

(19) $$\begin{array}{c} e_{p}=\sqrt{\left(e_{x}^{2}+e_{y}^{2}+e_{z}^{2}\right)}. \end{array}$$

Errors in paths for the test 3 were shown in Figures 5(e)–5(g), which ranged from 15.77 to +18.45 mm in *X*, from −15.55 mm to +15.91 mm in *Y*, and from 19.19 to +15.97 mm in *Z*, from the deviation of the reference path and the path in the TPAM, respectively. And in the OPAM, the errors ranged from −18.39 to +25.45 mm in *X*, from −20.55 to +29.68 mm in *Y*, and from −24.19 to +28.52 mm in *Z*. The mean absolute errors in TPAM were 3.31 mm in *X*, 3.35 mm in *Y*, and 3.70 mm in *Z* with standard deviations (SDs) of 0.21 mm, 0.32 mm, and 0.33 mm. Compared with the mean absolute errors in OPAM, 9.68 mm in *X*, 9.56 mm in *Y*, and 10.87 mm in *Z* with standard deviations (SDs) of 0.67 mm, 0.47 mm, and 0.43 mm, the errors in TPAM were very low. During the object manipulation tasks, some minor correction of the position and the orientation for overshoot was required, mainly preceding a gripper inserting the peg into the hole, as shown in Figures 5(b)–5(d).

The safe pipe was designed to detect the potential collision and the warning pipe constrained the robot EE to follow the referenced path. The robot EE should be in the safe pipe and the warning pipe. When the robot EE passed through the safe pipe, the system should give a warning to the operator to adjust his/her operation. Tables 1 and 2 gave the results of the pipes.

**Figure 6:**
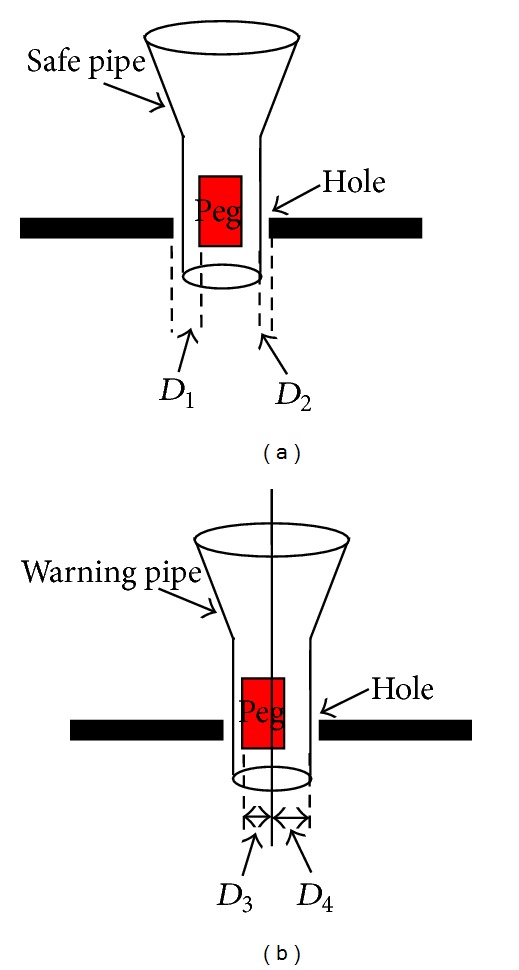
Definition of the distances. *D*_1_: the distance between the peg and the edge of the hole. *D*_2_: the distance between the pipe and the edge of the hole. *D*_3_: the sum of the radius of peg and the deviation of the peg from the reference path; *D*_4_: radius of the warning pipe.

The manipulation task required that the robot EE needs to get close to the table so that it could insert the peg into the hole. When the robot EE got close to the table, the safe pipe expanded to create more safe space to protect the robot EE without collision. In order to make sure the space inside of the safe pipe was safe, the obstacles should be outside of the safe pipe. When the safe pipe expanded and encountered the edge of the hole, it would stop to expand. So if the robot EE kept moving and passed through the safe pipe, it would collide with the edge of the hole.  Table 1 shows the results of the variety of the safe pipe and Figure 6 shows the definition of the distances. During the period from 1st s to 9th s, the robot EE got close to the hole continuously. From the 11th s, the robot started to insert the peg into the hole. At the 13th s, the distance between the robot EE and the obstacle was 0.8 mm, but it was 1.0 mm away from the safe pipe and the obstacle. At that time, the robot EE passed through the safe pipe and would collide with the obstacle and the system gave a warning to the operator. After the operator adjusted the operation (positions and orientation), the robot EE moved into the safe pipe again; it was 1.1 mm away from the obstacle which is larger than the distance (1.0 mm) between the safe pipe and the edge of the hole.

The purpose of the warning pipe was to detect the deviation from the reference path. When the robot EE deviated from the deviation, the system would predict the safe distance through the speed and the acceleration and change the radius of warning pipe. When the robot EE passed through the warning pipe, the system would give the operator warning. The initial purpose was that the warning pipe could detect the potential dangers and adjust the real path in time. The greater the deviation was, the larger the safe distance predicted was, and the radius of the warning pipe was smaller. During the manipulation proceeding, the robot EE should follow the reference. But due to muscle tremors or operate miss, the robot EE deviated from the reference some time.  Table 2 shows the results of the radius changes of the warning pipe. At the 13th s, the deviation was 8.7 mm, along with 8.3 mm in the radius of the warning pipe. So the system gave the warning to the operator to remind him to adjust the operation in time.

The main purpose of the experiments is to evaluate the improvement of the operator's manipulating with the virtual fixtures derived from complex geometry, compared with nonassisted instrument manipulation. Our constrained control method works for the traditional master-slave teleoperation. We evaluated the user's performance of peg-into-hole, with both OPAM and TPAM. We simply used an available PHANTOM DESKTOP as the teleoperation master hand controller.

The experiments were completed by 6 operators with both OPAM and TPAM. The mean absolute errors between the reference path and the robot path were shown in Tables 3 and 4. In OPAM mode, the mean absolute errors (MAEs) for the six tests in the period of approaching ranged from 9.24 mm to 12.44 mm in *X*, 8.21 mm to 9.90 mm in *Y*, and 9.56 mm to 13.90 mm in *Z*, and the mean errors (MEs) were 10.29 mm in *X*, 9.41 mm in *Y*, 12.11 mm in *Z*, and 18.56 mm in 3D path with standard deviations (SDs) of 1.06 mm, 0.57 mm, 1.68 mm, and 1.15 mm. MAEs in TPAM ranged from 2.72 mm to 4.49 mm in *X*, 1.68 mm to 5.53 mm in *Y*, and 3.70 mm to 4.89 mm in *Z*, and the MEs were 3.47 mm in *X*, 3.20 mm in *Y*, 4.06 mm in *Z*, and 6.32 mm in 3D path, with SDs of 0.35 mm, 1.46 mm, 0.20 mm, and 0.44 mm. In the period of inserting, the MEs in OPAM were 1.38 mm in *X*, 1.58 mm in *Y*, 1.62 mm in *Z*, and 2.69 mm in 3D path. Comparing with the OPAM, the MEs in path drop 1.47 mm in TPAM. The results show that the operating errors are lower in TPAM than those in OPAM, which is due to the assisted pipes making the manipulation more precise.

**Figure 7:**
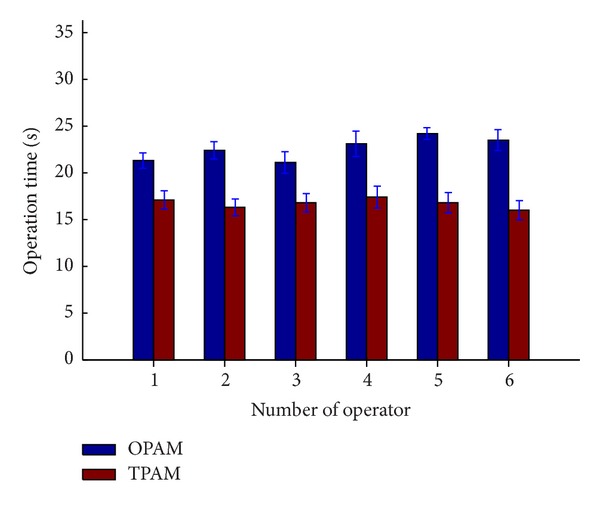
Operation time.

The operating time is longer in OPAM than in TPAM, as shown in Figure 7. Without the aid of the warning pipe, the operators needed to make more minor correction to adjust the orientation. The operating time in OPAM ranged from 21 s to 24 s with the average time being 22.56 s. Compared with the OPAM, the time drops to 16.8 s in TPAM, which ranged from 16 s to 18 s.

## 5. Conclusion

This paper has developed a real-time task-based control method of a telerobot in a precise interactive teleoperation environment. Computer guidance (remote teleoperative control) employing spatial motion constraints generated by virtual fixtures can assist the operators in skilled manipulation tasks. The virtual fixtures can provide the desirable properties, such as safety and collision avoidance.

The results of the experiments demonstrate that the TPAM is better in shortening the operating time and the accuracy improvement. The experimental results show that there is remarkable reduction in operating tension on avoiding collision of the instrument, which can improve the manipulation efficient.

In this paper, the comparison has been taken concerning the performance of OPAM and TPAM in a complicated working volume. The performance-comparison experiment results show that the TPAM operation is more intuitive for operator to use. The execution time with TPAM operation is shorter than OPAM, as it is also more precise than OPAM. The experiment comparison shown here is intended to demonstrate the improvement of our spatial constraints method in TPAM.

The primary focus of this paper is to develop a technique for controlling the motion of teleoperated robots via simple real-time geometric virtual fixtures. In the future work, we will use hybrid feedback patterns (force and vision) to assist the operator.

## Figures and Tables

**Table 1:** The results of the safe pipe.

Time	Joints values						*D*_1_/mm	*D*_2_/mm	PorN
	θ_1_/(°)	θ_2_/(°)	θ_3_/(°)	θ_4_/(°)	θ_5_/(°)	θ_6_/(°)			
11th	0.13	−94.25	−64.33	0.35	−94.45	−0.44	1.8	2.0	N
12th	0.16	−94.42	−64.14	0.41	−94.63	−0.16	1.3	1.0	N
13th	0.21	−94.41	−64.34	0.36	−60.46	−0.34	**0.8**	**1.0**	**Y**
14th	0.31	−94.31	−64.54	0.33	−60.42	−0.21	1.1	1.0	N
15th	0.41	−94.21	−64.23	0.31	−60.41	−0.13	1.5	1.0	N

PorN: pass through or not.

**Table 2:** The results of the warning pipe.

Time	Joints values						*D*_3_/mm	*D*_4_/mm	PorN
	θ_1_/(°)	θ_2_/(°)	θ_3_/(°)	θ_4_/(°)	θ_5_/(°)	θ_6_/(°)			
11th	0.13	−94.25	−64.33	0.35	−94.45	−0.44	7.7	9.3	N
12th	0.16	−94.42	−64.14	0.41	−94.63	−0.16	8.2	8.8	N
13th	0.21	−94.41	−64.34	0.36	−60.46	−0.34	**8.7**	**8.3**	**Y **
14th	0.31	−94.31	−64.54	0.33	−60.42	−0.21	8.4	8.6	N
15th	0.41	−94.21	−64.23	0.31	−60.41	−0.13	8.0	9.0	N

PorN: pass through or not.

**Table 3:** Mean absolute errors (MAEs) for 6 tests measured by the robot in the period of approaching.

Times	OPAM				TPAM			
	*X*/mm	*Y*/mm	*Z*/mm	Path/mm	*X*/mm	*Y*/mm	*Z*/mm	Path/mm
1	10.54 ± 0.89	9.87 ± 0.55	9.56 ± 0.56	17.32 ± 0.13	2.87 ± 0.32	1.68 ± 0.54	3.82 ± 0.34	5.07 ± 0.48
2	9.24 ± 0.76	9.90 ± 0.65	14.44 ± 0.65	19.80 ± 0.32	3.59 ± 0.33	2.20 ± 0.51	4.89 ± 0.27	6.45 ± 0.24
3	9.68 ± 0.67	9.56 ± 0.47	10.87 ± 0.43	17.41 ± 0.67	3.31 ± 0.21	3.35 ± 0.32	3.70 ± 0.33	5.99 ± 0.42
4	12.44 ± 0.88	8.21 ± 0.78	13.90 ± 0.21	20.38 ± 0.48	4.49 ± 0.13	3.31 ± 0.25	4.02 ± 0.38	6.88 ± 0.17
5	10.40 ± 0.32	9.49 ± 0.32	11.58 ± 0.97	18.23 ± 0.85	3.84 ± 0.42	3.16 ± 0.36	4.42 ± 0.45	6.46 ± 0.61
6	9.47 ± 0.56	9.43 ± 0.45	12.35 ± 0.77	18.20 ± 0.82	2.72 ± 0.24	5.53 ± 0.34	3.56 ± 0.44	7.12 ± 0.56
MEs	10.29 ± 1.06	9.41 ± 0.57	12.11 ± 1.68	18.56 ± 1.15	3.47 ± 0.35	3.20 ± 1.46	4.06 ± 0.20	6.32 ± 0.44

**Table 4:** Mean absolute errors (MAEs) for 6 tests measured by the robot in the period of inserting.

Times	OPAM				TPAM			
	*X*/mm	*Y*/mm	*Z*/mm	Path/mm	*X*/mm	*Y*/mm	*Z*/mm	Path/mm
1	1.54 ± 0.08	1.87 ± 0.05	1.56 ± 0.06	2.88 ± 0.03	0.87 ± 0.02	0.68 ± 0.04	0.82 ± 0.04	1.37 ± 0.08
2	1.24 ± 0.07	1.90 ± 0.06	0.94 ± 0.05	2.45 ± 0.02	0.59 ± 0.03	0.80 ± 0.01	0.89 ± 0.07	1.33 ± 0.09
3	1.68 ± 0.06	1.56 ± 0.04	1.87 ± 0.03	2.95 ± 0.07	0.71 ± 0.01	0.35 ± 0.02	0.70 ± 0.03	1.06 ± 0.08
4	1.44 ± 0.08	1.21 ± 0.07	1.90 ± 0.01	2.67 ± 0.08	0.99 ± 0.03	0.51 ± 0.05	0.82 ± 0.06	1.38 ± 0.07
5	0.90 ± 0.03	1.49 ± 0.03	2.08 ± 0.07	2.71 ± 0.05	0.84 ± 0.02	0.66 ± 0.06	0.42 ± 0.05	1.15 ± 0.09
6	1.47 ± 0.05	1.43 ± 0.04	1.35 ± 0.07	2.45 ± 0.02	0.72 ± 0.04	0.53 ± 0.04	0.56 ± 0.04	1.05 ± 0.07
MEs	1.38 ± 0.06	1.58 ± 0.57	1.62 ± 0.68	2.69 ± 0.65	0.79 ± 0.35	0.59 ± 1.46	0.70 ± 0.20	1.22 ± 0.44
